# Determinants of default from full completion of vaccination among children between 12 and 23 months old in Yilmana Densa district, west Gojam zone, Ethiopia, 2019

**DOI:** 10.3389/fpubh.2022.974858

**Published:** 2022-10-14

**Authors:** Desalegn Koyto Mekuria, Getachew Hailu, Melkamu Bedimo, Alemu Adela Tefera

**Affiliations:** ^1^District Health Office, Asagirt District, North Showa, Amhara, Ethiopia; ^2^Health Science College, Bahir Dar University, Bahir Dar, Ethiopia; ^3^Department of Biomedical Science, Health Science College, Debre Berhan University, Debre Berhan, Ethiopia

**Keywords:** determinant, default to vaccination, unmatched case-control, Yilmana Densa district, child vaccination

## Abstract

**Background:**

Vaccination is one of the best cost-effective public health interventions to safeguard children from vaccine-preventable diseases. In Ethiopia, the prevalence of default to the full completion of child immunization is high. However, the determinants of default to full completion have not been thoroughly investigated in this study area. Therefore, this study assessed the determinants of default to the full compilation of vaccination among children between 12 and 23 months old in Yilmana Densa District, west Gojam, northwest Ethiopia.

**Methods:**

A community-based unmatched case-control study design was employed in the Yilmana Densa district among 343 (111 cases and 232 controls) randomly selected 12–23 months old children. Face-to-face interviews were used to collect data using a multistage sampling method. For analysis, data were entered into epidata version 3.1 and exported to SPSS 23 software. Descriptive analysis followed by binary and multivariable logistic regression analysis was conducted. The statistical significance was declared at a *p*-value of 0.05.

**Result:**

This study identified that mothers who had not attended ANC follow-up [AOR = 5.55, 95% CI: (1.789–17.217)], mothers who had not gotten information about vaccinations [AOR = 8.589, 95% CI: (4.414–16.714)], and mothers whose time taken to reach vaccination site is more than 39 min were at higher risk to default from completion of vaccination [AOR = 3.252, 95% CI: (1.952–5.417)]. Furthermore, maternal waiting time (>45 min) for child vaccination [AOR = 2.674, 95% CI: (1.517–4.714)] and home delivery [AOR 3.19, 95% CI: (1.751–5.814)] were risk factors to default child from full completion of vaccination.

**Conclusion:**

Mothers delivered at home, mothers not attending ANC follow-up, mothers who did not get health information about the vaccine, mothers taking longer time to reach the vaccination site, and staying longer time for child vaccination are causes of default. Motivated institutional delivery services utilization is recommended. The district office should consider the distribution of vaccination sites by the opening of new outreach site to reduce the waiting time of mothers.

## Background

Vaccination is the administration of a mixed vaccine intended to activate a recipient's immune system to release antibodies that provide future protection against specific infectious diseases. Vaccination is a simple, safe, lifesaving, and cost-effective health intervention shown to reduce childhood morbidity and mortality (1). Vaccination significantly minimizes vaccine-preventable diseases worldwide after the implementation of the expanded immunization program in 1974 (2). All countries are recommended to reach ≥90% vaccination coverage for all vaccines by 2020 (3). However, large magnitudes of children are not fully vaccinated as recommended by the WHO. According to WHO guidelines, children are considered fully vaccinated when they received the 10 vaccines against the 10 vaccine-preventable diseases: Bacillus Chalmette Guerine (BCG), three doses of pentavalent, two doses of Rota vaccine, diphtheria pertussis and tetanus, hepatitis B hemophilus influenza type b (DPT-Hep B-HIB), pneumococcal conjugated vaccine (PCV), and three doses of polio vaccine and measles vaccine by the age of 12 months (4).

The dropout rate is measured between the first and third doses of the DPT-Hep B-HIB vaccine. The proportion of the first dose Diphtheria-Pertussis-Tetanus (DTP) received to complete the third dose of DTP is varied among World Health Organization (WHO) regions. Despite the variation, the 3-dose DTP series immunization program's effectiveness in 2016 was <5% and only a few countries achieved the plan due to different barriers (5, 6).

Globally, about 5% (6.6 million) of children drop out, but the highest third dose of DTP vaccine dropout rate was recorded in the African Region, which accounts for 11% (3.1 million) and the lowest dropout rate was in Western Pacific Region, at 0.4% (0.08 million) (7). In the year 2016, worldwide, 14% of children failed to complete the series of three doses of DTP. However, 19.5 million children failed (dropout) to receive the DTP3 dose. Among those who did not receive the third dose of DTP, 11.8 million (61%) were in 10 countries (2, 8). Nigeria (18%), India (16%), Pakistan (7%), Indonesia (6%), and Ethiopia (4%) are the top five defaulting rate countries (7, 9).

Defaulting vaccination is a continued problem in all African regions, including Ethiopia, putting children at serious risk of potentially fatal vaccine-preventable diseases. Demographic barriers such as parents' lack of education, low socioeconomic status, populations living in difficult-to-reach areas challenged the success of EPI. Additionally, programmatic difficulties, including vaccine stock-outs and violence, continue to hinder some children from benefiting from comprehensive immunization at the local or national levels. Program expenses and a lack of political will also contribute to the issues (7, 10).

In Ethiopia, due to the presence of childhood vaccine trends, infant, child, and under-five mortality have been steadily declining over the last 18 years. However, there is the problem of coverage, full completion of vaccination, and vaccination schedule timeliness. Regional coverage of the third dose of DTP, OPV3, and the first dose of the measles-containing vaccine (MCV_1_) remained stable in 2016 (11), according to WHO/UNICEF Estimates of National Immunization Coverage released in July 2017. On the other hand, the 2016 EDHS report showed that vaccination coverage was 73% for DTP1, 53% for DTP3, 56% for Polio3, and 54% for measles. But, only 39% of full completion vaccination coverage has been reported (12).

According to the EDHS (2016) report, Ethiopian children are vaccinated for the first doses of vaccination but not the consecutive doses, which leads to a high dropout rate in Ethiopia. Therefore, about 1.5 million children died every year due to a high dropout rate (3). Therefore, this study aimed to determine factors associated with incomplete immunization coverage among children between 12 and 23 months old in Ethiopia, Yilmana Densa district of West Gojam zone.

## Methods and materials

### Study design

An unmatched community-based case-control study design was used.

### Study area and period

This study was conducted in Yilmana Densa, West Gojam zone, Ethiopia, from 30 March to 15 May 2019. Yilmana Densa district is the second-most populated district in the west Gojam Zone with an estimated number of 275,187 populations. Of which, 136,218 (49.5%) were female and 5,200 (5.1%) were children between 12 and 23 months of age. The district has 5 urban and 30 rural kebeles. EPI is provided by all health centers, health posts, and outreach sessions. Health service coverage was 89%. According to the 2017/18 district health office report, the full completion vaccination coverage was 66%, which is below the WHO standard (80%).

### Population

All children between 12 and 23 months of age, and who had started at least one dose of the routine immunization program in the Yilmana Densa district were the source population. All children between 12 and 23 months of age residing in randomly selected kebeles were the study population.

### Eligibility criteria

#### Cases

All children aged 12–23 months lived in the study area for the past 2 years and the children at least received one vaccination exposure.

#### Controls

All children aged 12–23 months have completed all the recommended vaccines.

#### Exclusion criteria

Children whose parents or guardians struggle to communicate immunization information about their child succinctly.

### Sample size calculation and sampling technique

The sample size was calculated using EPI info version 7.2.1.0 and is based on the following assumptions: A power of 80% with a 95% confide level (CL), a maximum tolerable error of 5%, and the one case to two control (1:2) ratio with Odds ratio of 5.7. With a 10% non-response rate, 345 people were the estimated sample size (115 cases and 230 controls). Proportions of maternal health service utilization among cases (97.7%) and controls (88.1%) were obtained from the previous literature (13).

The study participants were selected using a stratified sampling technique. The district was classified into two strata: urban and rural residents. Then, one urban and seven rural kebeles were chosen at random to provide valid study subjects. Cases and controls in the kebeles were identified using child vaccination cards and a vaccine registration book from the health posts. Cases were children aged 12–23 months who have missed at least one dose from the recommended schedule (except for polio zero). The total sample size of 345 (115 cases and 230 controls) was allocated proportionally to each selected kebele. Finally, the study participants were selected randomly by the lottery method from all the selected kebeles and households. A data extraction checklist form was used to extract secondary data. Primary data were collected using a structured and interviewer-administered questionnaire.

### Study variables

Defaulting from full completion of vaccination (Yes/No) were the dependent variables. Whereas, the independent variables were socio-demographic factors such as age of mother, pregnancy status, residency, caretaker, maternal occupation, sex of the child, birth order, child's father, and paternal behavior; government-related factors: health budget policy and vaccine demand/vaccine supply; health service accessibility: time taken to get health post, waiting time for vaccination, place of delivery, antenatal care service, vaccine mode of transport to health facility/outreach site, and stock out vaccination place/time; maternal health service utilization: ANC/PNC, TT vaccination, antenatal conference participation, and inconvenient vaccination place/time, waiting time, appointment, and getting of health education about vaccination.

### Operational (working) definition

- **Complete vaccination:** A child aged 12 and 23 months who had received 10 basic vaccines.
- **Vaccination defaulters:** When a child missed at least one dose from the recommended schedule.
- **ANC follow-up of last pregnancy:** A woman who receives routine health services during her pregnancy in accordance with WHO recommendations of at least four visits for low-risk pregnancy.
- **Caregiver:** It refers to the most responsible person who provides child care for a child aged between 12 and 23 months whose biological mother is unable to provide intimate care.

### Data collection tools and procedure

Secondary data were extracted using a data extraction checklist form. Face-to-face interviews were used to collect primary data using a structured and pretested Amharic version questionnaire. The questionnaire was first prepared in English and then translated to Amharic. The questionnaire had designed to measure socio-demographic characteristics, maternal health service utilization, health facility access, paternal behavior, vaccination status of a child, and reasons for defaulting from full completion of vaccination. Children's vaccination cards were used to collect information about their vaccination status.

### Data quality assurance

The questionnaire was pretested on 5% of the study participants in another area. Data collectors and supervisors each received 1 day of training to ensure that they all had a common understanding of the study's objectives and each of the questionnaire's questions. Daily, data were checked for completeness, consistency, accuracy, and clarity. Communication with data collectors, supervisors, and principal investigators was maintained throughout the study period. Before data entry, the data collectors, supervisors, and principal investigator checked the returned collected data for completeness.

### Data analysis

The data were cleaned, coded, and entered into Epi data version 3.1 and exported to SPSS version 23 for analysis. The simple frequency with percentage, figure, and tables were used to display the descriptive part of the result. A bivariable logistic regression model was used to identify the determinant variables. A variable with *p* ≤ 0.2 in bivariable logistic regression was eligible for the multivariable logistic regression analysis model to control the confounding effect. Both bivariable and multivariable logistic regression models were used to identify the determinant factors of default to full completion vaccination. Odds ratio (OR) with a 95% confidence interval was used to identify the strength of associations. A *p* < 0.05% was considered a statistically significant association.

## Results

### Socio-demographic characteristics

A total of 343 respondents participated in this study with a response rate of 98%. Of these, 96 respondents in cases (87%) and 222 respondents in controls (95.7%) were biological mothers. The mean ± SD participants' age was 29.9 ± 6.8 years with a minimum age of 19 years and a maximum age of 51 years. Of these, 46 (41%) respondents of cases and 96 (39%) respondents of controls were between 25 and 34 years. Among all the participants, 88 cases (79%) and 178 controls (77%) were rural duelers. Ninety-two percent (102) cases (92%) and 227 controls (98%) were married. Eighty-eight cases (79%) and 173 controls (75%) were farmers, 85 cases (77%) and 150 controls (65%) were Illiterate, 91 (82%) cases and 161 controls (69%) were not currently engaged in women development army network (Table 1).

**Table 1:** Maternal and child Socio-demographic characteristics contributing factor of defaulting from completion of vaccination among children aged 12–23 months, in Yilmana Densa district, west Gojam, Ethiopia, 2019.

**Variables**	**Category**	**Cases**		**Controls**	
		**Frequency**	**Percent**	**Frequency**	**Percent**
Respondent	Care	15	13.5	10	4.3
	Mother	96	86.5	222	95.7
Place of residence	Rural	88	79.3	178	76.7
	Urban	23	20.7	54	23.3
Marital status	Married	102	92	227	97.8
	Not married	3	2.7	1	0.4
	Divorce	5	4.5	4	1.7
Maternal occupation	Farmer	88	79	173	74.6
	House wife	13	12	36	15.5
	Govt worker	2	2	11	5
	Trader	5	5	11	5
Maternal educational level	Illiterate	85	76.6	150	64.7
	Literate	26	23	82	35
Sex of the child	Male	66	60	120	52
	Female	45	41	112	48
Father and child	Not live together	24	22	15	6.5
	Live together	87	78	217	93.5
Family size	<5	78	70	156	67
	≥5	33	30	77	33

### Socio-demographic factors on defaulting of full completion of vaccination

Children with caregivers [COR: 3.469 (CI: 1.505–7.996)], illiterate mothers [COR: 1.787 (CI: 1–2.992)], farmer parents [COR: 1.787 (1.232–2.992)], and children who are not live together with their fathers [COR: 3.991 (CI: 1.999–7.968)] were more likely of default full completion of vaccination (Table 2).

**Table 2:** Association between maternal and child socio demographic characteristics and defaulting vaccination status among children aged 12–23 months, in Yilmana Densa district, West Gojam, Ethiopia, 2019.

**Variables**	**Case, *n* (%)**	**Control *n* (%)**	**COR (95% CI)**	***P-*Value**
**Respondent**				
Care giver	15 (13.5%)	10 (4.3%)	3.469 (1.505–7.996)	0.003
Mother	96 (86.5%)	222 (95.7%)	1	
**Place of residence**				
Rural	88 (79.3)	178 (76.7)	0.862 (0.497–1.494)	0.679
Urban	23 (20.7)	54 (23.3)	1	
**Maternal occupation**				
Farmer	88 (79)	173 (74.6)	1.787 (1.032–2.992)	0.027
House wife	13 (12)	36 (15.5)		
Gov't worker	2 (2)	11 (5)		
Trader	5 (5)	11 (5)		
**Maternal educational level**				
Illiterate	85 (76.6)	150 (64.7)	1.787 (1–2.992)	0.027
Literate	26 (23)	82 (35)	1	
**Sex of the child**				
Male	66 (60)	120 (52)	0.731 (0.462–1.155)	0.203
Female	45 (41)	112 (48)	1	
**Father of child**				
Not live together	24 (22)	15 (6.5)	3.991 (1.999–7.968)	0.001
Live together	87 (78)	217 (93.5)	1	

COR, Crude odds ratio; CI, Confidence interval.

### Health facility-related factors of defaulting full completion of vaccination

In the bivariate regression model, three variables (travel time, waiting time, and site of delivery) were found to be determinants of defaulting from full completion of vaccination. Children delivered at home were more likely to default from being fully vaccinated than those who were born at a health facility [OR: 3.156 (1.912–5.425)]. Primary caretakers who travel for more than 39 min have a higher risk of being defaulting vaccinated than those who travel for <39 min for child vaccination (OR: 3.221, 95% CI: 1.912–5.425). Primary caretakers with poor knowledge about the schedule of vaccination were more likely to incomplete the recommended vaccination of children (OR: 3.90, 95% CI: 1.60–9.51). Mothers/caretakers who waited for more than 45 min toward vaccinating their child were three times more likely to have a default from full completion of vaccinated children than those who waited <45 min [OR: 3.008 (1.678–5.394)] (Table 3).

**Table 3:** Association between heaths related factors and defaulting vaccination status of children.

**Variables**	**Case, *n* (%)**	**Control *n* (%)**	**COR (95% CI)**	***P-*Value**
**Travel time (in minute)**				
<39	43 (39)	151 (65)	2.948 (1.847–4.706)	< 0.001
≥39	68 (61)	81 (35)		
**Waiting time (in minute)**				
<45	74 (67)	178 (77)	1.648 (1.001–2.713)	0.034
≥45'	37 (33)	54 (23)		
**Site of delivery**				
Home	35 (32)	29 (13)	3.224 (1.845–5.634)	<0.001
Health facility	76 (68)	203 (87)		
**Mode of travel to vaccine site**				
On foot	111 (100)	216 (93)	0.661 (0.611–0.714)	0.611
By vehicle	0	16 (7)		

COR, Crude odds ratio; CI, Confidence interval.

### Maternal health service utilization related to defaulting of complete vaccination

Mothers who did not use any type of ANC service before delivery of the child were three times more likely to have defaulter children than mothers who did use ANC services [OR = 2.521 (95% CI: 2.521 (1.308–4.86)]. Mothers who did not have vaccination-related health information were nine times more likely to have defaulter children than mothers who had gotten child vaccination-related health information {OR = 8.589 (4.414–16.714)} (Table 4).

**Table 4:** The effect of Maternal and child health services on factors and defaulting from completion of child immunization in Yilmana Densa district, North West of Ethiopia, 2019.

**Variables**	**Category**	**Default vaccination**	**Fully vaccinated**	**COR (95% CI)**	***P-*Value**
Attended ANC	No	27 (24)	5 (2)	14.593 (5.441–39.14)	<0.001
	Yes	84 (76)	227 (98)		
Received TT vaccine	No	40 (36)	20 (9)	5.661 (3.129–10.239)	<0.001
	Yes	71 (64)	211 (91)		
Getting information from HW	No	77 (69)	30 (13)	14.684 (8.446–25.53)	<0.001
	Yes	34 (31)	201 (87)	1	
Pregnancy conference	No	86 (78)	124 (53)	3.219 (1.901–5.45)	<0.001
	Yes	23 (21)	106 (46)	1	
Attending ANC.	≤2	48 (56)	53 (24)	4.235 (2.498–7.178)	<0.001
	>2	37 (44)	173 (77)		
	TT once	20 (29)	48 (23)	1.3605	0.203
TT_received	2 and above	49 (71)	160 (77)		

COR, Crude odds ratio; CI, Confidence interval.

### Reasons for defaulting from full completion of child vaccination

**Figure 1:**
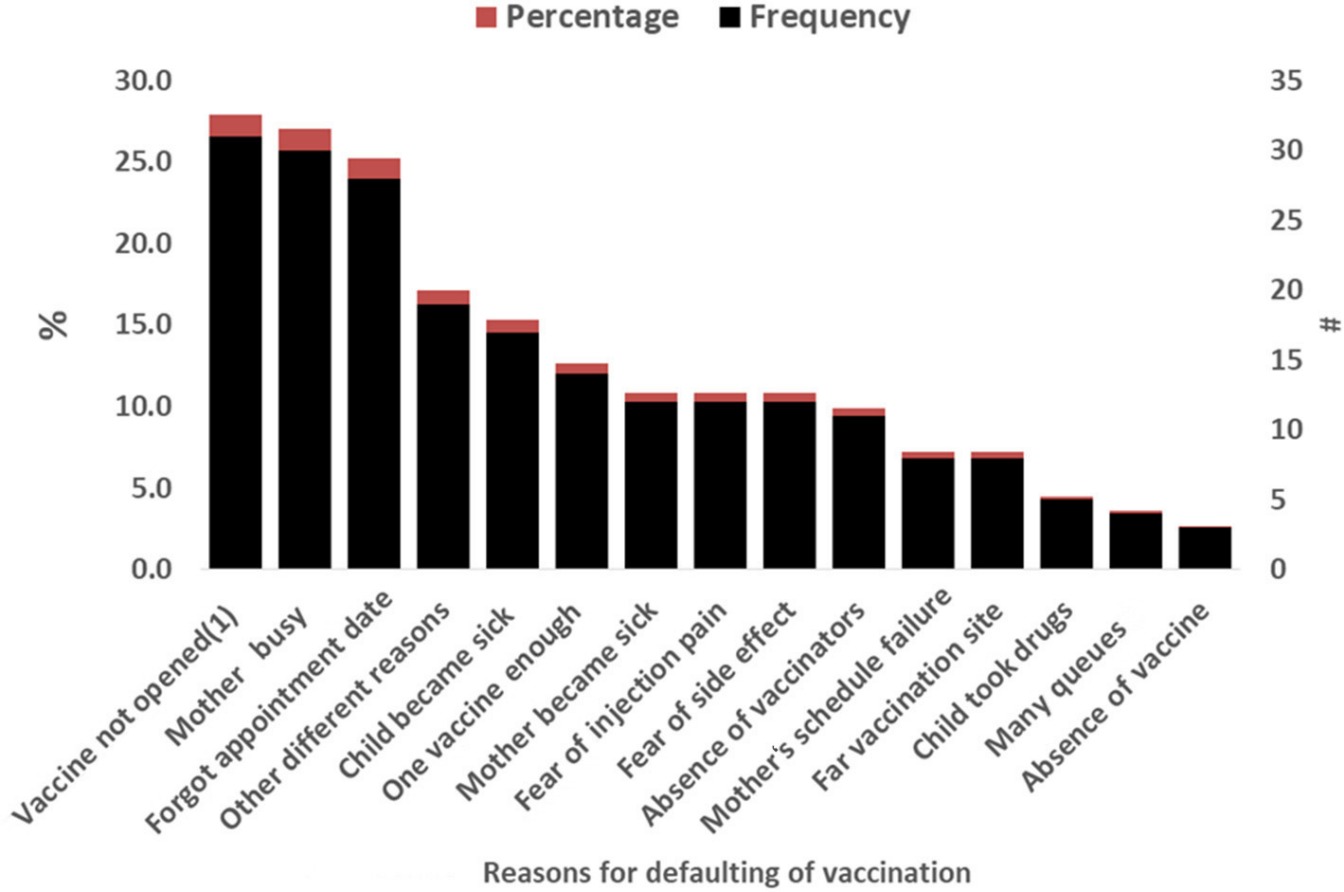
Reasons a child defaulting from full completion of vaccination among respondents in Yilmana Densa District, West Gojam zone, northwest of Ethiopia, 2019.

The main causes for defaulting from full completion of vaccination were the vaccine vials cannot be opened for one child, reported in 31 of 111 cases (28%), mother's becoming busy with her own routinely activity reported in 30 of 111 (27%), and forgetfulness of vaccination schedules/appointment, reported in 28 of 111 cases (25%) (Figure 1).

### Independent predictors of incompletion of immunization

From the multiple logistics regression analysis, after controlling the confounding variable, 14 variables such as respondent status, maternal education level, the status of child's father, site of birth, the time taken to vaccination site, waiting time for vaccination, attended ANC follow-up in current pregnancy, maternal Tetanus toxoid (TT) vaccination status, and maternal participation in the conference during the current pregnancy period were found to be independent predictors of defaulting vaccination.

Babies born at home were more likely to remain defaulting vaccination than those who were delivered at a health institution (AOR: 3.156, 95% CI: 1.912–5.425). Children living with a single partner had a higher risk of being incompletely vaccinated than those living with both partners [AOR: 3.68, 95% CI: (1.806–7.508)]. Mothers/caregivers who did not get information about the vaccination were nine times more likely to default from full completion of vaccination of their children than those who had gotten information about vaccination [AOR: 8.589, 95 % CI: (4.414–16.714)]. Children with primary caregivers who take more than 39 min to arrive at the vaccination site are more likely to skip full-course vaccination [AOR = 3.252 (1.952–5.417)]. Similarly, primary caregivers of children who wait for vaccination for <45 min are less likely to default on vaccination than those who wait more than 45 min for vaccination of their children [AOR = 2.674 (1.517–4.714)]. Furthermore, children with mothers who did not attend ANC during their previous pregnancy [OR: 5.55, 95% CI: (1.789–17.217)] were six times more likely to skip vaccinations than children with mothers who tried to attend ANC care during their previous pregnancy. The mother's educational level was significantly related to vaccination defaulting. Children who were born from illiterate mothers were two times at a higher risk to default from completion of vaccination than children who were born from educated mothers (Table 5).

**Table 5:** Multiple logistic regression analysis of Independent predictors of defaulting from full completion of child vaccination in Yilmana Densa district, North West, Ethiopia, May 2019.

**Variables**	**Case, *n* (%)**	**Control *n* (%)**	**95% CI**		
			**Crude OR**	**Adjusted OR**	***P-*Value**
**Respondent status**					
Care giver	15 (14)	10 (4)	3.469 (1.50–0.003)	3.229 (1.345–7.753)	0.009
Mother	96 (86)	222 (96)	1	1	
**Maternal educational level**					
Illiterate	85 (76.6)	150 (65)	1.787 (1.787–2.99)	1.725 (1.006–2.959)	0.048
Literate	26 (23)	82 (35)	1	1	
Child of father			1	1	
Not live to gather	24 (22)	15 (6.5)	3.991 (1.999–7.968)	3.68 (1.806–7.508)	<0.001
Live together	87 (78)	217 (94)	1	1	
**Time taken to reach vaccination site (in minute)**					
≥39'	68 (61)	81 (35)		3.252 (1.952–5.417)	<0.001
<39'	43 (39)	151 (65)	1	1	
**Waiting time for vaccination (in minute)**					
≥45'	37 (33)	54 (23)	1	2.674 (1.517–4.714)	0.019
<45'	74 (67)	178 (77)		1	
**Maternal site of current delivery**					
Home delivery	35 (32)	29 (13)	3.224 (1.845–5.634)	3.156 (1.912–5.425)	<0.001
Health facility delivery	76 (68)	203 (87)	1	1	
**Attended ANC follow up**					
No	27 (24.3)	5 (2.2)	14.593 (5.441–39.14)	5.55 (1.789–17.217)	0.003
Yes	84 (75.7)	227 (97.8)	1	1	
**Mothers received TT vaccine during current pregnancy**					
No	40 (36)	20 (8.6)	5.661 (3.129–10.239)	2.447 (1.179–5.076)	0.016
Yes	71 (64)	211 (91)	1	1	
**Maternal getting information about vaccination**					
No	77 (69)	30 (13)	14.68 (8.44–25.53)	8.589 (4.414–16.714)	<0.001
Yes	34 (30.6)	201 (86.6)	1	1	
**Maternal participate pregnancy conference**					
No	86 (77.5)	124 (53)	3.219 (1.901–5.45)	2.191 (1.262–3.804)	0.005
Yes	23 (20.7)	106 (45.7)	1	1	
**ANC attending**					
Less than ≤2	48 (56.5)	53 (23.5)	4.235 (2.498–7.178)	2.521 (1.308–4.86)	<0.006
More than >2	37 (43.5)	173 (76.5)	1	1	

AOR, Adjusted odds ratio; CI, Confidence interval.

## Discussion

In Ethiopia as well as the Amhara region, tremendous activity was done to reduce child mortality but in recent time, the outbreaks of different diseases become a public health problem.

This study assessed predictors of vaccination default and revealed that maternal education status, time taken to reach vaccination sites by walking, ANC follow-up, primary caretakers who received information from HEW about child vaccination, maternal participation in current pregnancy conference, mothers taking TT vaccine, primary care status, site of delivery, and living time of child without a father were independent predictors of vaccination default of child after controlling for all other variables. This study identified that maternal (caregivers) educational status was an independent determinant factor to complete full immunization. This finding was agreed with other studies conducted in Ethiopia and abroad (14–17). This finding can be explained by the fact that educated mothers may have better knowledge and attitudes about the benefits and requirements of child immunization than uneducated mothers (caregivers). In this study, the time taken to the vaccination site was an independent determinant factor of defaulting to vaccination. Other studies in Pakistan, Ethiopia Uganda, and Indonesia also reported similar results (18–21). This finding was not surprising given that traveling to distant locations takes time and money for mothers and caregivers. This may force mothers to abstain from vaccinating their children.

This study also revealed that children with mothers who had not received the TT vaccine during their most recent pregnancy were two times more likely to drop out from full completion of vaccination as compared with mothers who had received TT vaccination during their most recent pregnancy. This study agreed with studies conducted in Ethiopia and other parts of the world (22–27). The possible explanation may be that mothers who received TT vaccination have the chance to get vaccination-related information during their visit for TT vaccination. This study also identified that the place of delivery was the determinant factor in vaccine defaulting. Other studies also reported that children who delivered at home were at higher risk of defaulting than children who delivered at the health facility (19, 28–30). This is due to mothers who delivered at the health facility could have a better understanding of the need for timely and full immunization of children. In this study, ANC follow-up was another determinant factor for defaulting. This study was consistent with other studies reported in Ethiopia and Kenya (18, 19, 29, 30). ANC is a means to deliver adequate information about child vaccination to mothers.

In this study, children of mothers who have not received health information from HEWs were at higher risk of default vaccination. Other studies here in Ethiopia also reported similar results (17, 24). This finding indicated that HEWs are central to delivering information about child vaccination. This study also identified that defaulting of vaccination were higher in the children of mothers who did not participate in pregnancy conference. This finding implies that it is critical to raise mothers' vaccination awareness through locally appropriate mechanisms such as HEW visits, pregnancy conference participation, and ANC. Perhaps the content of vaccination health education should be reviewed.

## Limitation of the study

The mothers'/caretakers' economic status was not investigated, which may impact default vaccination. Recall bias was introduced because mothers may forget about their children's vaccinations and other issues.

## Conclusion

This study found that respondent status (non-biological), maternal educational level, the presence of the child's father with the child, and maternal participation in health development army network status were significant among socio-demographic determinants for defaulters of child vaccination. Health facility factors such as the site of delivery, travel time, and waiting time also had a significant association. Additionally, health service utilization factors including ANC follow-up, TT-receiving status, obtaining of health information during ANC follow-up, and maternal participation in pregnancy conferences were the determinant factors of defaulting vaccinations.

To minimize defaulting vaccination, ANC follow-up and mothers' participation in pregnancy conferences should be strengthened. The health professionals and the health extension workers should give special attention to children who live with caregivers and not with their fathers. District health offices should increase the distribution of vaccination and vaccination sites to address district areas that are far from health centers and health posts. Further studies concerning the issue of the timeliness of children's vaccinations and dropout tracing mechanisms are recommended.

## Data availability statement

The raw data supporting the conclusions of this article will be made available by the authors, without undue reservation.

## Ethics statement

Verbal informed consent was approved by the institutional review committee, Bahir Dar University College of Medicine and Health Sciences Institutional Review Board. Ethical clearance was obtained from the Institutional Review Board (IRB) of Bahir Dar University, College of Medicine and Health Sciences. This study was conducted as per the Declaration of Helsinki. Permission letters were obtained from the Yilmana Densa district health office. Verbal informed consent was obtained from each study participant before the actual data collection begins. The Privacy and confidentiality of the study participants were maintained throughout the study.

## Author contributions

All authors listed have made a substantial, direct, and intellectual contribution to the work and approved it for publication.

## Conflict of interest

The authors declare that the research was conducted in the absence of any commercial or financial relationships that could be construed as a potential conflict of interest.

## Publisher's note

All claims expressed in this article are solely those of the authors and do not necessarily represent those of their affiliated organizations, or those of the publisher, the editors and the reviewers. Any product that may be evaluated in this article, or claim that may be made by its manufacturer, is not guaranteed or endorsed by the publisher.

## Abbreviations

AAC: antenatal care
ARCC: Africa Regional Certification Committee
BCG: Bacillus Calmette–Guerin
DTP: Diphtheria-Tetanus-Pertussis
DTa: Diphtheria-Tetanus
EDHS: Ethiopian Demographic and Health Survey
EPI: Expanded Program on Immunization
Epi-Info: Epidemiological Information's
GVAP: Global vaccine action plan
HEW: health extension worker
HBV: Hepatitis B Vaccine
HF: Health Facility
Hib: Homophiles influenza type B disease
IPV: Injectable Polio Vaccine
MCV: Measles Containing Vaccine
MMR: Measles Mumps Rubella
MOH: Ministry of Health
OPV: Oral Polio Vaccine
PCV: Pneumococcal Conjugate Vaccine
Rota: Rota Virus Vaccine
SPSS: Statistical Package for Social Science
VPDS: Vaccine preventable disease.
